# Studies of Myc super-competition and clonal growth in *Drosophila* males and females

**DOI:** 10.17912/micropub.biology.000502

**Published:** 2021-12-09

**Authors:** Abigail J Svoysky, Jeffrey L Bellah, Laura A Johnston

**Affiliations:** 1 Department of Genetics and Development, Columbia University Medical Center

## Abstract

Cell competition is a cell selection process that arises in growing tissues as a result of interactions between cells of different fitness. This behavior is also observed in Myc super-competition, where healthy wild type cells in growing wing discs of *Drosophila* are outcompeted by nearby cells that express higher levels of the Myc oncogene. Most work on Myc super-competition has examined it in mixed populations of male and female larvae. However, as physiological and genetic differences between *Drosophila *males and females could affect the competitive behavior of cells, we have investigated whether sex differences affect the process. Here we show that both male and female wing disc cells are subject to Myc super-competition. Female disc cells appear to be more sensitive to competitive elimination than male cells, potentially due to differences in baseline cellular Myc levels between the sexes. We also report sexual dimorphism of cell size and number between male and female growing wing discs that is independent of competition; wing discs and wing pouches from females are larger than males’ due to larger cell size and cell number. We suggest that separately examining male and female tissues in cell competition assays could enhance our understanding of the effects of sex-specific pathways on cell and super-competition.

**Figure 1. Female-male differences in somatic clonal growth and cell competition:**
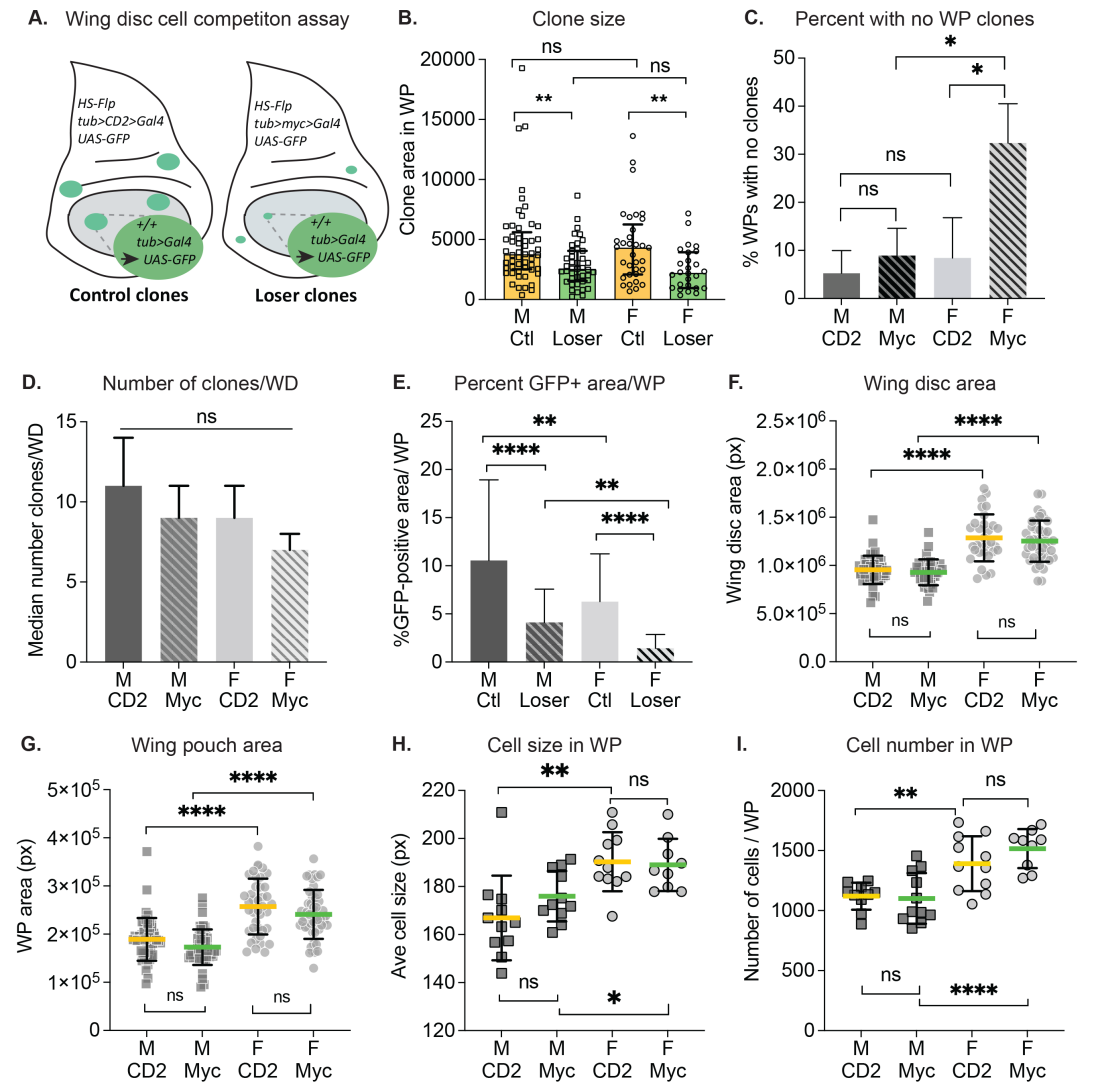
**(A). In vivo assay for Myc induced super-competition.** See text and methods for details. **(B). Cell competition occurs in both males and females.** The size of control (Ctl) and Loser clones, each marked by expression of GFP, was scored in wing discs (WD) from male (M) and female (F) larvae (n=41-50 WD per genotype). Loser clones from both M and F were significantly smaller than their respective controls. Graph shows the area in pixels of clones in the wing pouch (WP) region (oval region in **A**). **(C).** **Female wing discs subject to cell competition infrequently contain WP clones.** Shown is percent of WPs from M and F wing discs that were completely devoid of clones. **(D).** **The total number of clones per wing disc is similar in both sexes.** Graph shows the median number and 95% CI of total number of clones per wing disc for each genotype. **(E). Total GFP-positive area per WP in female discs is reduced compared to male discs.** The percentage of the WP covered with GFP+ cells was calculated by dividing the GFP-positive area over WP area. GFP-positive WP coverage is reduced in F in both competitive and noncompetitive contexts. **(F-I).** **Whole wing discs and WP regions from females are significantly larger than those from males, due to increased cell size and cell number.** Whole wing disc **(F)** and WP **(G)** sizes were measured for M and F of each genotype. **(H). Cell size in the female WP is larger than in males.** Thenumber of nuclei within three independent 100 x 100-pixel squares in the WP of M or F wing discs was counted and averaged to estimate cell size. Nuclei were stained with DAPI. In both competitive and noncompetitive contexts, females had significantly larger cells than males. **(I) Cell number in the female WP is larger than in males.** To determine the number of cells per WP, each WP area was divided by the corresponding average cell size calculated in **(H)**. Females had greater numbers of cells per WP than males. All data in this figure is derived from the same data set (n= 3 biological replicates). In panels **(H-I**), data is from 1 replicate of 9-11 WDs. In each panel, unpaired t-tests with Welch’s correction were used to determine statistical significance. Error bars in **B** represent median and interquartile range; error bars in **C** and **E-I** represent mean ± standard deviation. Ns = not significant, * p < 0.05, ** p < 0.01, ***p<0.001, ****p<0.0001.

## Description

Cell competition is a cell selection process that arises in growing tissues as a result of interactions between cells of different fitness (Simpson, 1979). A well documented example of this behavior is Myc super-competition, where healthy wild type (WT) cells in the growing wing discs (adult wing primordia) of *Drosophila* larvae are outcompeted by nearby cells that express higher levels of the growth factor Myc (de la Cova*et al.* 2004; Moreno and Basler, 2004). Short-range signaling interactions between the cell populations cause the WT cells, termed “losers”, to be eliminated via apoptosis, allowing the Myc-expressing cells to colonize the tissue (de la Cova*et al.* 2004; Senoo-Matsuda and Johnston, 2007; Meyer*et al.* 2014; Alpar*et al.* 2018). Most of the existing work on cell competition has examined it in mixed larval populations of males and females without accounting for sex differences. However, physiological and genetic differences between *Drosophila* males and females could affect the behavior of cells during cell competition. We have investigated cell competition in separated male and female larvae, using Flp recombinase-mediated excision of a gene cassette expressing the transcription factor Myc (*>myc>Gal4*; competitive context) or the cell marker CD2 (>*CD2>Gal4*; non-competitive context) to generate loser and control clones, respectively. A short heat shock induces the Flp recombinase, which stochastically excises the *myc* or *CD2* gene cassette from a few cells in the developing wing disc. Each excision event is heritable and generates a clone marked by the expression of GFP under Gal4 control **(Fig. 1A)**. Excision of the *>myc>* cassette yields a cell and its progeny that lack the extra *my*c, but still express endogenous *myc*; however, this clone is surrounded by fitter cells that still have the >*myc>* cassette. Thus, the GFP-positive clonal cells become “losers”, while the surrounding cells with the *>myc>* cassette become “winners”. Excision of the >*CD2>* cassette serves as a control because its loss has no measurable effect on cell fitness (de la Cova*et al.* 2004). After a defined growth period, loser clone size is compared to control clone size generated in parallel in the non-competitive context. Competitive loser-winner interactions lead to death of loser cells, thereby reducing loser clone size (de la Cova*et al.* 2004; Meyer*et al.* 2014; Alpar*et al.* 2018).

To determine how cell competition differs between male and female larvae, we compared loser and control clone sizes from male and female wing discs in a cell competition assay (de la Cova*et al.* 2004) **(Fig. 1A)**. We specifically measured clones contained within the wing pouch (WP) region, where competition is strongest (Alpar*et al.* 2018). In both males and females, loser clones were significantly smaller than their respective control clones, indicating that cell competition occurs in wing discs from both male and female larvae **(Fig. 1B)**. However, we noticed that a disproportionate number of wing discs from females in the competitive context contained no clones in the WP at all. We calculated the percentage of WPs with no clones in male and female discs **(Fig. 1C)**, and found that the baseline no-clone rate among noncompetitive male and female WPs, as well as male competitive WPs, was 7.5% on average. In contrast, 32.7% of female WPs from the competitive context had no clones. To investigate this further, we counted the total number of clones in whole wing discs from both contexts and compared clone numbers between males and females. Whole disc clones were counted to minimize interference from the more severe competitive effects in the WP (Alpar*et al.* 2018). Overall, the number of clones present in the discs at the end of the growth period was not significantly different in male versus female wing discs **(Fig. 1D)**.

Adult *Drosophila* females are larger than males (Alpatov, 1930; Testa*et al.* 2013), and this size difference is also apparent in larval wing discs **(Fig. 1F-G)**. To account for disc size differences, we calculated the percentage of GFP-positive clonal area within each WP for male and female wing discs. This metric was restricted to GFP-positive cells within the WP, even if portions of the clones continued outside of the region. As expected, in both males and females from the competitive environment, GFP-positive WP coverage was considerably reduced compared to WP coverage from the non-competitive cohorts **(Fig. 1E, Loser vs Ctl)**. Surprisingly, female wing discs, from both competitive and noncompetitive contexts, had significantly less GFP-positive coverage of the WP compared to males of either context **(Fig. 1E)**. To further investigate growth differences between male and female discs, we compared the number and size of cells in the WP of male and female discs **(Fig. 1H-I**). The cells in female WPs were slightly, but significantly, larger than male WP cells, in both the competitive and noncompetitive contexts **(Fig. 1H)**. In addition, the number of cells per WP was greater in females than males, in both genetic contexts **(Fig. 1I)**. Consequently, the larger size of the female WP can be attributed to both larger cells and greater numbers of cells.

What are the mechanisms underlying the sexual differences in susceptibility to cell competition? One possibility, at least in the Myc super-competition system used here, is the difference in baseline Myc levels between females and males. The endogenous *myc* gene is on the X chromosome, so females have two copies while males have only one. Dosage compensation in *Drosophila* upregulates genes on the male X chromosome to approximately match female levels (Conrad and Akhtar, 2012); however, the *myc* gene does not undergo dosage compensation (Mathews*et al.* 2017). As a result, females have roughly twice the baseline expression of *myc* than males (Mathews*et al.* 2017). In the >*myc>* larvae used here, the additional copy of *myc* introduced in the cassette maintains the difference in Myc levels between males and females, such that females have three *myc* copies while males have two. Because female сells express higher *myc* levels, it is possible that after excision of the cassette the deviation in *myc* expression is easily recognized by surrounding cells, allowing vigorous out-competition of loser cells from the female WP. Conversely, because males express less *myc* overall, cells that deviate below the baseline level may be less susceptible to competition and eliminated more slowly in the male disc.

In summary, our study indicates that male and female wing disc cells are both subject to Myc super-competition. Compared to males, we observed that a significantly higher fraction of female wing discs in the competitive context were completely devoid of clones, suggesting an increase in susceptibility to cell competition in females. We demonstrate that female wing discs are larger than male discs, confirming previous studies (Mathews*et al.* 2017). In addition, we report sexual dimorphism of cell size between males and females in the growing larval wing discs. This result is consistent with findings that adult female flies have larger fat body cells and wing cells than do males (Alpatov, 1930; Rideout*et al.* 2015). Notably, on average, clone size was not different between males and females within each particular genotype, arguing that the clonal growth rate – which reflects cell proliferation rates – is similar in males and females. This suggests that the sexually dimorphic disc size existed from an early larval stage, prior to clone induction (~48h after egg laying). In this light, it is interesting that the median number of clones in the entire wing disc trended lower in females than in males **(Fig.1D)**. The cohort of males and females for each clonal context derive from the same parents and are thus genetically identical, implying a similar Flp-out excision rate in both sexes. However, we observed no significant difference in the number of clones per disc between males and females, despite the finding that female discs likely had more cells at clone induction and would be expected to yield more clones. Thus, it is possible that the Flp-out excision rate was higher in males than females. A higher Flp-out excision rate in males would also explain our surprising finding that the GFP-positive coverage was significantly greater in male than female WPs **(Fig. 1E)**. Alternatively, the Flp-out excision may occur with similar rates in males and females, but the DNA repair required after the excision may be less successful in females, leading to rapid death of the excised cells. Future experiments will be useful in parsing out these possibilities. We suggest that examining male and female tissues independently in cell competition assays could enhance our understanding of the effect of dosage compensation and other sex-specific pathways on the outcomes of competing cell populations.

## Methods

*Clonal Assays.* Flies in experimental crosses were raised at 25°C on cornmeal-molasses Fly Food R (LabExpress, Ann Arbor, MI). Flies were allowed to lay eggs for 2 hour periods on grape-agar plates supplemented with fresh yeast (Sigma, St. Louis, MO) paste in a moist environment at 25°C. After 24 hours, freshly hatched first instar larvae were transferred to cornmeal-molasses food supplemented with fresh yeast paste and allowed to develop at 25°C. To induced clones, second instar larvae were heat shocked 48 hours after egg laying (AEL) at 37°C for 7 min (for larvae containing the *tub>CD2>Gal4* cassette) or 13 min (for larvae carrying the *tub>myc>Gal4* cassette), as in (Alpar *et al.* 2018). Larvae were then allowed to grow at 25°C until 92 hours AEL, when they were isolated, divided by sex based on ovary or testis detection, and dissected for fixation. Dissections were performed in 1X phosphate buffered saline (PBS) at room temperature (RT). Anterior, inverted halves of dissected larvae were fixed with 4% paraformaldehyde (PFA) in 1X PBS for 20 minutes at RT on a nutator and then washed 3 times with 1X PBS containing 0.1% Tween 20 (PBTw) (Sigma, St. Louis, MO) for 5 minutes each at RT. Anterior half-carcasses were stained with 500 μL of 1 μg/mL of the DNA stain DAPI in PBTw for 10 minutes at RT and washed twice with PBTw for 5 minutes. Wing discs were dissected from the fixed larval carcasses at RT in 1X PBS, mounted in 15 μL of VectaShield (Vector Laboratories, Burlingame, CA) on glass slides and covered with 18×18 mm cover slips.

*Image Acquisition and Processing.* Wing discs were imaged with the Zeiss Imager.M2 (White Plains, NY) and Zen Pro software using a 20X objective lens with 0.8 numerical aperture and ApoTome.2. Images were captured as either a Z stack of 1 μm increments encompassing the entire wing pouch, or as images with the wing pouch in focus (without ApoTome). Clones in the wing disc were marked by expression of nuclear GFP. Clone areas were measured using the wand tool in Fiji. Measurements were taken and reported in pixel (px) units. Wing pouch areas were measured using the free-form drawing tool in Fiji. Images were blinded and scrambled prior to scoring. The number of clones per disc was determined by counting clones in all focal planes of the disc using the Zeiss Imager.M2 microscope. The WP region was demarcated by the first dorsal fold and first ventral fold on either side of the dorsal/ventral compartment boundary, as well as the first lateral folds along the anterior/posterior axis of the disc (Alpar *et al.* 2018).

*Calculation of cell size and cell number*. Cell size was estimated by counting the number of nuclei (stained by DAPI) in a 100 by 100-pixel square in the WP region of wing discs, in triplicate, at 92 hr AEL. In Fiji, squares were placed on dorsal, ventral, and medial-lateral regions of each wing pouch image to account for regional size differences of cells. Average cell size in pixels was calculated by dividing 10,000 px^2^ by the average number of nuclei per square.

*Statistical Analysis.* Prism 8 (GraphPad Software, San Diego, CA) was used to generate all plots. Clone sizes were plotted showing individual data points with median and interquartile range. Competitive and noncompetitive contexts were compared pairwise among each genotype using unpaired *t*-tests with Welch’s correction. The percentage of wing pouches devoid of clones was calculated for each genotype; percentages were plotted, showing mean with standard deviation. Percentages were compared among genotypes using unpaired *t*-tests with Welch’s correction. Mean percent GFP+ area, wing disc size, wing pouch size, wing pouch cell size, and number of cells per pouch were plotted with standard deviation, and values were compared among genotypes using unpaired *t*-tests with Welch’s correction. Median number of clones per wing disc was plotted with 95% confidence interval for each genotype, and values were compared using unpaired *t*-tests with Welch’s correction.

## Reagents

**Table:** 

Fly Food R	(LabExpress, Ann Arbor, MI)
Dry yeast	(Sigma, #YSC2)
DAPI	(Molecular probes, #D1306)
VectaShield mounting medium	(Fisher, #NC9532821)
***Drosophila* strains:**	**Reference:**
*y w hsflp122; +; +*	(Alpar *et al.* 2018)
*w; tub>myc>Gal4/CyODfd-Yfp; +*	(Alpar *et al.* 2018)
*w; tub>CD2>Gal4/ CyODfd-Yfp; +*	(Alpar *et al.* 2018)
